# Editorial: Insights into falls efficacy and fear of falling

**DOI:** 10.3389/fragi.2025.1644435

**Published:** 2025-11-13

**Authors:** Shawn Leng-Hsien Soh, William R. Young, Tianma Xu

**Affiliations:** 1 Health and Social Sciences Cluster, Singapore Institute of Technology, Singapore, Singapore; 2 Public Health and Sport Sciences, University of Exeter, Exeter, England

**Keywords:** falls efficacy, fear of falling, concerns about falling, balance confidence, balance recovery confidence, fall prevention and management, measurement instruments, rehabilitation

## Introduction

Falls efficacy and fear of falling (FOF) are key psychological constructs influencing mobility, independence, and quality of life among older adults (Soh, 2022; Merchant et al., 2020). Although conceptually related, these constructs are distinct: falls efficacy is a cognitive construct referring to the perceived ability to prevent and manage falls (Soh et al., 2024), while FOF relates to vigilance to threats and resultant emotional reponses (Lee and Tak, 2023). Recognising this nuance is essential for clinicians and researchers, who must be equipped to select appropriate measurement tools aligned with the targeted construct and to tailor interventions accordingly. This Research Topic presents five insightful studies that collectively deepen our understanding of falls efficacy and fear of falling, advancing our perspectives on their conceptualisation, measurement, and implications for clinical practice.

## Disentangling falls efficacy and FOF

The longstanding conflation of falls efficacy and FOF has posed challenges for clinicians and researchers. Early work by Hadjistavropoulos et al. (Hadjistavropoulos et al., 2011) clarified their distinction through a review of scales such as the Falls Efficacy Scale (FES) (Tinetti et al., 1990), the Activity-specific Balance Confidence (ABC) Scale (Powell and Myers, 1995), and the Survey of Activities and Fear of Falling in the Elderly (SAFE) Scale (Lachman et al., 1998). Falls efficacy was conceptualised as distinct from fear of falling, aligning more closely with balance confidence. Building on this, Ting et al. examined the convergent and predictive validity of the ABC, the Balance Recovery Confidence (BRC), and the Falls Efficacy Scale-International (FES-I) scales among community-dwelling older adults. Their findings revealed moderately strong correlations between ABC and FES-I, with moderate alignment with the BRC. Both ABC and BRC predicted concerns about falling, offering further evidence that these constructs, while related, are not interchangeable. Ting et al.’s work provides a valuable contribution to furthering insights into conceptual boundaries and the need to consider appropriate assessment tools.

## Disentangling fear and concerns of falling

Takla et al. offer additional conceptual clarity between FOF and concern about falling (CAF) by introducing the Concern and Fear of Falling Evaluation (CAFFE) Scale. A psychometrically validated instrument that positions CAF and FOF along a continuum adds nuances to how individuals experience fall-related psychological states. The CAFFE provides a promising direction for future research and an assessment tool to evaluate psychologically informed interventions. This aligns with recent commentary by Ellmers and colleagues (Ellmers et al., 2023), who highlighted the importance of differentiating adaptive and maladaptive concerns about falling. While CAF may promote protective behaviours, it can also become maladaptive, manifesting as hypervigilance, activity avoidance, or even denial of risk. This highlights the importance of careful assessment and tailored intervention strategies.

## Awareness of potential discrepancies between psychological constructs and other factors

Gaining insight into fall efficacy, FOF, and/or CAF is a useful starting point for crafting person-centred approaches in fall prevention and management practice. Two studies highlighted the potential for discrepancies between psychological constructs and other factors, such as fall risk, warranting greater attention to this aspect. Choudhury et al. explored the interplay between habitual physical activity and the alignment or misalignment of perceived and physiological fall risk in community-dwelling older adults using the Fall Risk Appraisal (FRA) framework. Individuals were categorised into four groups: Low FOF and high physiological fall risk (“Incongruent”); Low FOF and low physiological fall risk (“Rational”); High FOF and high physiological fall risk (“Congruent”); and High FOF and low physiological fall risk (“Irrational”). The study highlighted that FOF is a distinct barrier to activity engagement and underscores the need for tailored interventions.

Inoue et al. examined discrepancies in perceived fall risk between physical therapists and stroke patients in a rehabilitation hospital. The study found that patients consistently underestimated their fall risk, particularly those who went on to experience multiple falls during hospitalisation. Such underestimation advocates the need to enhance tailored patient education and generate greater self-awareness in post-stroke care.

Ultimately, clinicians and researchers are keen to find ways to identify individuals at risk of falling so that prevention strategies can be implemented. Wapp et al. presented a robust fall rate prediction model using prospective fall data from the Swiss CHEF cohort. Through count regression modelling, they identified that the history of falls and FOF were the strongest predictors of future fall rates. Their findings advocate for integrating psychological factors into fall risk assessments to enhance personalised fall prevention strategies. A summary of the five articles is listed in Table 1.

**TABLE 1:** Summary of the five articles.

First Author	Year of Publication	Assessment Tool(s) Used	Sample Size	Studied Population	Intervention	Main Findings
Takla et al.	2024	CAFFE (Concern and Fear of Falling Evaluation)	1,025	Individuals with multiple sclerosis (MS) in the US	N/A	The CAFFE scale is reliable and valid for distinguishing between concern about falling (CAF) and fear of falling (FOF) in MS patients, with defined tipping points for transitioning from CAF to FOF.
Ting et al.	2025	Activities-specific Balance Confidence (ABC) Scale, Balance Recovery Confidence (BRC) Scale, Falls Efficacy Scale-International (FES-I)	131	Community-dwelling older adults in Singapore	N/A	ABC and BRC scales measure distinct but related constructs. ABC is a stronger predictor of concerns about falling (FES-I) than BRC. Tools should be selected based on the specific construct of interest
Wapp et al.	2022	Falls Efficacy Scale-International (FES-I), Timed Up and Go (TUG), Four Stage Balance Test (FSBT), Forward Reach Test, Five Times Sit-To-Stand, gait speed, Older People’s Quality of Life Questionnaire (OPQOL-35)	353	Community-dwelling older adults in Switzerland	Three home-based exercise programs (Test and Exercise, Otago, Helsana)	Prior falls and fear of falling (FES-I) are strong predictors of future falls. Recurrent fallers (≥4 falls) are at highest risk. The final prediction model included prior falls and FES-I score
Inoue et al.	2023	Falls Efficacy Scale–International (FES-I), Functional Independence Measure (FIM), Stroke Impairment Assessment Set-motor function (SIAS-m)	426	Patients with subacute stroke admitted to a rehabilitation hospital in Japan	Fall prevention measures (e.g., wrist bands, supervision, environmental adjustments, education)	Patients consistently underestimated their fall risk compared to physical therapists. Discrepancies in fall risk perception decreased in non-fallers and single fallers but persisted in multiple fallers. The FES-I assessed by therapists correlated more strongly with motor and ADL scores than patient self-assessments
Choudhury et al.	2024	Short Falls Efficacy Scale–International (FES-I), BTrackS Balance System, 30-s sit-to-stand test, ActiGraph GT9X accelerometer (MIMS metric)	163	Community-dwelling older adults aged 60+ in the US	N/A	FOF is a significant barrier for older adults to participate in high-intensity physical activities, regardless of their balance and strength. Physical activity programs for older adults should develop tailored intervention strategies (cognitive reframing, balance and strength exercises, or both) based on an individual’s FOF and physiological fall risk

## Future directions

Future research needs to incorporate falls efficacy and FOF/CAF into fall prevention and management practice. We anticipate that the levels of these psychological constructs may be different between various clinical populations, including those with neurological, musculoskeletal, and cardiopulmonary conditions, as well as seniors with varying physical abilities residing in hospitals, residential facilities, or those living independently in the community. A robust psychometric evaluation of relevant measurement instruments is necessary to ensure these tools are suitable for the targeted populations. Interventions must be refined based on individual psychological profiles, taking into account contextual influences such as environmental and cultural factors. Longitudinal studies will be essential to understanding how changes in falls efficacy and FOF/CAF could influence long-term mobility, independence, and fall outcomes. A broader integration of psychological metrics into clinical fall risk assessments can help optimise personalised strategies for older adults.

## Conclusion

These contributions have deepened our understanding of two critical fall-related psychological dimensions. The topic, “Insights into Falls Efficacy and Fear of Falling,” highlights the importance of nuanced conceptualisation, measurement, and intervention, moving beyond one-size-fits-all approaches toward tailored, person-centred care. With validated tools and emerging theoretical models, the field is well-positioned to address both the cognitive and emotional dimensions of fall risk. We hope this Research Topic catalyses further innovation and collaboration in fall prevention research and practice. We thank all contributing authors, reviewers, and readers for advancing this important field.
